# Incidence of delayed gastric conduit emptying in patients undergoing esophagectomy: a systematic review and meta‑analysis

**DOI:** 10.1007/s10388-025-01133-8

**Published:** 2025-05-28

**Authors:** Jonathan Sivakumar, Qianyu Chen, Cuong Phu Duong

**Affiliations:** 1 Department of Upper Gastrointestinal Surgery, Peter MacCallum Cancer Centre, 305 Grattan St, Melbourne, VIC 3000 Australia; 2 Department of Surgery, The University of Melbourne, Melbourne, Australia

**Keywords:** Delayed gastric conduit emptying, Esophagectomy, Incidence

## Abstract

Delayed gastric conduit emptying (DGCE) is a significant and prevalent complication following esophagectomy, adversely affecting recovery and quality of life. The true burden of DGCE remains uncertain due to considerable variability in reported incidence rates. This study aimed to determine the incidence of DGCE following esophagectomy and how this is impacted with varying diagnostic criteria. A systematic review was conducted across major databases, including PubMed, MEDLINE, Embase, Web of Science, and Cochrane, to identify studies reporting the incidence of DGCE following esophagectomy. The incidence rates were pooled and analyzed using a random-effects model, with subgroup analyses for potential sources of heterogeneity such as pyloric interventions, conduit dimensions, and anastomotic height. Among 5176 screened records, 125 studies met the eligibility criteria. The pooled incidence of early DGCE was 15.9% (95% CI 11–21%), and late DGCE was 9.4% (95% CI 7.1–11.9%). Significant heterogeneity was observed across studies, driven by variations in diagnostic methods. Subgroup analysis indicated that prophylactic pyloric drainage was not associated with a statistically significant effect on early DGCE (OR 0.76; *p* = 0.38) or late DGCE (OR 0.71; *p* = 0.44). DGCE represents a significant burden for esophageal cancer survivors, with considerable variability in its reported incidence, underscoring the urgent need for a standardized diagnostic criterion. The adoption of the recently published international consensus definition is crucial for reducing the heterogeneity, as well as improving the identification and management of DGCE.

**Supplementary Information:**

The online version contains supplementary material available at 10.1007/s10388-025-01133-8.

## Introduction

The main curative treatment modality for esophageal cancer (OC) is tumor resection, with or without perioperative chemotherapy or chemoradiation. Esophagectomy is a major operation which involves OC resection with radical lymphadenectomy, followed by mobilization and tubularization of the remnant stomach to create an esophago-gastric anastomosis in the thoracic cavity [1, 2]. As OC survivorship has increased, there has been a growing emphasis on functional status and health-related quality of life following esophagectomy.

Delayed gastric conduit emptying (DGCE) is major contributor to impaired quality-of-life for post-esophagectomy patients, with an incidence of up to 50% [3–5]. This clinical entity is characterized by impaired motility of the gastric conduit, leading to nausea, vomiting, early satiety, and reflux symptoms [6, 7]. While the pathophysiology of DGCE is not completely understood, it is thought to be primarily related to division of the vagus nerve, the neural pathway necessary for effective gastric peristalsis and pyloric sphincter relaxation [5, 8]. The diagnostic criterion for DGCE has varied between institutions and in published literature. To address this issue, a more structured definition of DGCE was proposed at an international consensus meeting [9].

The true burden of DGCE remains uncertain, with considerable inconsistency in how this outcome is reported. This review aims to determine the incidence of DGCE and examine its variation across the different diagnostic definitions.

## Methods

### Search strategy

A comprehensive review of the literature was undertaken through to October 2024 to identify articles pertaining to the incidence of DGCE. The PubMed, Medline, Embase, Web of Science, and Cochrane Library electronic databases were systematically searched from inception. The following medical subject heading (MeSH) terms were used as a minimum in combination with Boolean operators and free-text terms, applied in multiple different combinations: “Delayed gastric conduit emptying”, “Gastric conduit dysfunction”, “Gastroparesis”, “Functional complications”, “Oesophageal cancer surgery”, and “Esophagectomy”. A complete search strategy for a single database defined with all keywords and subject headings is included (Electronic supplementary material, Table S1). The search strategy was supplemented by manually screening the references of relevant published studies. Screening of articles and their selection was performed independently by two authors (J.S., Q.C.). Disagreements at any point of this process were solved by consensus or by consulting a third reviewer.

### Eligibility criteria

Abstracts of all the retrieved studies were screened to determine need for full-text review. The detailed examination of remaining full-text articles was undertaken to ascertain their suitability for inclusion in the review based on whether they reported on delayed gastric conduit emptying. The studies were selected if they met the following inclusion criteria: (a) reported the incidence of delayed gastric conduit emptying; (b) original paper with independent data; (c) published as a full-text article in a peer-reviewed journal. No language restrictions were applied. The studies were excluded according to the following criteria: (a) data based on colonic or jejunal interposition; (b) redundant data duplicated or partially duplicated in selected publications of the same cohort; (b) data specific to pediatric patients; (c) incomplete data; (e) reports on animal models; and (f) case reports, editorial letters, reviews and conference abstracts.

### Data extraction

Following a screen of abstracts to filter out studies outside of the review criteria, a detailed examination of full-text articles of the remaining studies was undertaken to ascertain their suitability for inclusion in the review. Data extraction was performed by two authors (J.S., Q.C.). The recorded data included author information, study design, study period, type of procedure, definition of DGCE, allocation into early or delayed DGCE, whether an intra-operative pyloric intervention was performed, type of gastric conduit and level of esophago-gastric anastomosis. The reporting of DGCE incidence was categorized into *early DGCE* if onset occurred during the index admission or within the first 14 days post-operatively, while all other cases were classified as *late DGCE*. In the absence of explicit temporal documentation, cases were conservatively designated as *late DGCE* by default. Incidence was reported for each outcome of interest, including comparisons between potential sources of heterogeneity–prophylactic pyloric intervention, conduit dimensions, and anastomosis height.

### Quality assessment

A comprehensive critical appraisal of the included studies was conducted to ensure methodological rigor and the reliability of findings. The Cochrane Risk of Bias 2 (RoB 2) tool was applied for randomized controlled trials (RCTs) to examine five domains that address key aspects of trial design, implementation, and outcome reporting [10]. The Risk of Bias in Non-Randomized Studies of Interventions (ROBINS-I) tool was employed for cohort and case–control designs to evaluate seven domains that systematically cover potential confounding factors, methodological limitations in participant selection, and deviations from intended interventional protocols [11]. Any disagreements between reviewers during the appraisal process were resolved through discussion and, where necessary, consultation with a third author to achieve consensus.

### Statistical analysis

The incidence data were extracted as proportions, with the total number of patients developing DGCE as the numerator and the total number of patients undergoing esophagectomy as the denominator. Pooled incidence from included studies was calculated Freeman–Tukey transformation adjusting for single proportion. Pooled odds ratio (OR) was calculated using random-effects restricted maximum likelihood method. To compare rates of early and late DGCE depending on different anastomotic heights, conduit dimensions or whether prophylactic pyloric drainage was performed, two-sample *Z* test for proportions was used to compare two incidences, and Marascuilo’s procedure was used to compare multiple proportions. Inter-study heterogeneity was evaluated using both the *I*^2^ and *H*^2^ statistics. Tau-squared (*τ*^2^) values were also calculated to estimate between-study variance in a random effects model, with values closer to zero indicating less variability. Funnel plots and Egger’s tests were used to assess for publication bias. A 95% confidence interval (CI) was calculated to present the overall pooled estimate. Statistical significance was accepted at a *p* value of 0.05. Stata18.5 was used for statistical analysis.

### Standards of reporting

This systematic review and meta-analysis was conducted in accordance with the Preferred Reporting Items for Systematic Reviews and Meta-Analyses (PRISMA) statement checklist and Assessing the Methodological Quality of Systematic Reviews (AMSTAR) guidelines [12, 13]. The review protocol was registered in the International Prospective Register of Systematic Reviews (PROSPERO) database (CRD42024577878) on 19 th August 2024 [14].

## Results

### Study selection and characteristics

**Fig. 1:**
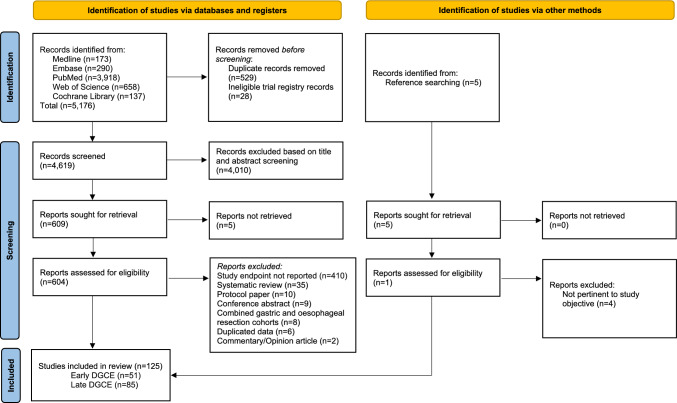
Flow diagram for systematic review. Source: Page MJ, et al. BMJ 2021;372:n71. https://doi.org/10.1136/bmj.n71. This work is licensed under CC BY 4.0. To view a copy of this license, visit

The systematic search yielded 5176 records from all databases, of which 529 duplicate entries and 28 ineligible trial registry records were removed prior to screening. Following title and abstract screening, 4010 records were excluded for irrelevance, leaving 609 full-text articles assessed for eligibility. Five additional studies were identified through reference searches. Of the 614 total articles evaluated, 125 studies met the inclusion criteria and were included in this review, with 51 studies focusing on early delayed gastric conduit emptying (DGCE) and 85 on late DGCE (Fig. 1).

The included studies were published between 1990 and 2024, with the characteristics summarized in Table 1. The studies were predominantly retrospective cohort studies (*n* = 76), and the sample sizes ranged from 8 to 1294 patients, with variable follow-up periods from 30 days to 7 years among the 68 studies that reported this.

**Table 1:** Characteristics and outcomes of studies reviewed

Author	Country	Study design	Study type	Surgical technique	Sample size	Follow-up period	Classification	Definition of early DGCE	Incidence of early DGCE	Definition of late DGCE	Incidence of late DGCE
Abdelrahma 2024	UK	Cohort study	Retrospective	Esophagectomy; Ivor-Lewis	100	100% follow-up to 5 years	Early and late	Modified Delphi process consensus	20/100 (20%)	Need for post-operative pyloric dilatation beyond 14 days (median 3 months; range 0–13 months)	12/100 (12%)
Antonoff 2014	USA	Cohort study	Retrospective	Esophagectomy; transthoracic (56.0%), transhiatal (44.0%)	293	47.8% follow up to beyond 12 months	Late	–	–	Need for post-operative pyloric dilatation long-term	38/293 (13%)
Babic 2022	Germany	Cohort study	Prospective	Esophagectomy; Ivor-Lewis	816	–	Early	Modified Delphi process consensus	226/816 (27.7%)	–	–
Bagheri 2014	Iran	RCT	Prospective	Esophagectomy; approach not specified	60	–	Early	Routine upper gastrointestinal contrast swallow study at day 7 post-operatively	15/60 (25.0%)	–	–
Benedix 2017	Germany	Cohort study	Retrospective	Esophagectomy; transthoracic	182	100% Follow up to beyond 9 months	Early	Any of the following: (1) > 7 day requirement for NGT, (2) > 3 day use of erythromycin, (3) gastric conduit obstruction/dilatation on post-operative barium swallow or endoscopy	71/182 (39.0%)	–	–
Bolger 2023	Canada	Cohort study	Prospective	Esophagectomy; approach not specified	171	–	Early and late	Both of the following: (1) subjective clinical evaluation of DGCE; (2) need for post-operative pyloric dilatation during index admission	16/171 (9.4%)	Both of the following: (1) subjective clinical evaluation of DGCE; (2) need for post-operative pyloric dilatation long-term	11/171 (6.4%)
Bolton 2013	USA	Cohort study	Retrospective	Esophagectomy; approach not specified	120	–	Early	Subjective clinical evaluation of DGCE—criteria not detailed	20/108 (18.5%)	–	–
Boshier 2018	UK	Cohort study	Retrospective	Esophagectomy; two-stage (76.0%), three-stage (24.0%)	100	–	Early	Routine upper gastrointestinal contrast swallow study at day 4–5 post-operatively	35/100 (35.0%)	–	–
Brunner 2023	Germany	Cohort study	Prospective	Esophagectomy; Ivor-Lewis	70	100% Follow-up to 2 years	Late	–	–	Modified Delphi process consensus	19/70 (27.1%)
Cerfolio 2009	USA	Cohort study	Prospective	Esophagectomy; Ivor-Lewis	221	Mean follow-up of 40 months	Early	Routine upper gastrointestinal contrast swallow study at day 4 post-operatively	186/221 (84.1%)	–	–
Chang 2017	China	Cohort study	Retrospective	Esophagectomy; approach not specified	1294	–	Early	Subjective clinical evaluation of DGCE—typical symptoms at day 7–14 post-operatively	19/1294 (1.5%)	–	–
Chen 2013	China	Cohort study	Retrospective	Esophagectomy; McKeown	142	Median follow-up of 26 months (Range 6–57 months)	Late	–	–	Subjective clinical evaluation of DGCE—Criteria not detailed	6/142 (4.2%)
Chen 2021	Taiwan	Cohort study	Retrospective	Esophagectomy; Ivor Lewis (39.4%); McKeown (60.6%)	526	100% follow-up to 3 years	Late	–	–	Subjective clinical evaluation of DGCE post-index admission—Criteria not detailed	61/526 (11.6%)
Datta 2014	USA	Cohort study	Retrospective	Esophagectomy; approach not specified	189	100% follow-up to 6 months	Late	–	–	Any of the following: (1) Post-operative clinical documentation, (2) Requiring pharmacologics for nausea/vomiting, (3) Abnormal gastric emptying scintigraphy with nausea/vomiting	43/189 (22.8%)
Deana 2021	Italy	Cohort study	Retrospective	Esophagectomy; approach not specified	110	100% follow-up to 12 months	Late	–	–	Esophagectomy Complications Consensus Group—Timeframe not detailed	1/110 (0.9%)
Decker 2020	USA	Cohort study	Retrospective	Esophagectomy; approach not specified	63	–	Early	Routine upper gastrointestinal contrast swallow study as inpatient prior to commencing oral intake	16/63 (25.4%)	–	–
Deldycke 2015	Belgium	Cohort study	Retrospective	Esophagectomy; Ivor-Lewis	322	100% follow-up to death date or study period completion	Early	Routine upper gastrointestinal contrast swallow study at day 5 post-operatively	119/322 (37.0%)	–	–
Deng 2010	China	Cohort study	Prospective	Esophagectomy; approach not specified	78	Median follow-up of 12 months (Range 8–60 months)	Early	Routine scintigraphic meal study at day 14 post-operatively (DGCE = > 10% Retention of meal in stomach at 4 h post-ingestion)	4/78 (5.1%)	–	–
Desprez 2020	France	Cohort study	Prospective	Esophagectomy; approach not specified	9	100% Follow up to beyond 6 months	Late	–	–	Pyloric distensibility < 10 mm2/mmHg based on EndoFLIP measurement beyond 6 months post-esophagectomy	6/9 (66.7%)
Ding 2021	China	Cohort study	Retrospective	Esophagectomy; approach not specified	25	100% Follow up to 5 years	Late	–	–	Criteria not detailed	1/25 (4%)
Djerf 2015	Sweden	Cohort study	Prospective	Esophagectomy; Ivor-Lewis	11	100% Follow up to beyond 2 years	Late	–	–	Radionuclide-labeled gastric scintigraphy at 2 years post-operatively	0/11 (0.0%)
Doran 2014	UK	Cohort study	Retrospective	Esophagectomy; Ivor-Lewis (100.0%)	207	–	Late	–	–	Need for post-operative pyloric dilatation long-term	13/207 (6.2%)
Eldaif 2014	USA	Cohort study	Prospective	Esophagectomy; Ivor Lewis; McKeown; transhiatal	322	100% Follow up to beyond 6 months	Early	Routine upper gastrointestinal contrast swallow study at day 5–7 post-operatively	30/322 (12.4%)	–	–
Elliot 2017	Ireland	Cohort study	Prospective	Esophagectomy; two-stage (69.2%); three-stage (23.1%); transhiatal (7.7%)	14	93% Follow up to beyond 3 months	Late	–	–	Criteria not detailed	0/14 (0%)
Ericson 2013	Sweden	Cohort study	Retrospective	Esophagectomy; approach not specified	195	–	Late	–	–	Need for post-operative pyloric dilatation long-term	13/195 (6.7%)
Feenstra 2023	Nether-lands	Cohort study	Prospective	Esophagectomy; approach not specified	65	100% Follow up to 30 days	Early	Routine upper gastrointestinal contrast swallow study at day 3–4 post-operatively	8/65 (12.3%)	–	–
Findlay 2015	UK	Cohort study	Retrospective	Esophagectomy; left thoracoabdominal (74.2%), Ivor Lewis (15.9%), three stage (2.3%), transhiatal (4.6%)	132	–	Late	–	–	Need for post-operative pyloric dilatation	13/132 (9.8%)
Finley 1995	Canada	Cohort study	Retrospective	Esophagectomy; Transhiatal (74.2%), Transthoracic (18.3%), thoracoabdominal (7.5%)	295	96% Follow up at 3 months	Early	Routine upper gastrointestinal contrast swallow study at day 7 post-operatively	16/295 (5.4%)	–	–
Fok 1991	Hong Kong	RCT	Prospective	Esophagectomy; Lewis Tanner (100%)	200	Mean follow-up of 17 months	Early and late	Routine upper gastrointestinal contrast swallow study at day 10 post-operatively	7/97 (7.2%)	Subjective clinical evaluation of DGCE—Criteria not detailed	13/200 (6.5%)
Forshaw 2006	UK	Cohort study	Prospective	Esophagectomy; left thoracoabdominal (100.0%)	38	–	Early	Routine upper gastrointestinal contrast swallow study at day 6 post-operatively	2/38 (5.3%)	–	–
Fransen 2023	Nether-lands	Propensity-matched analysis	Prospective	Esophagectomy; transhiatal (78.7%), transthoracic (62.9%)	1225	100% Follow up at 3 months	Late	–	–	Esophagectomy Complications Consensus Group—Timeframe not detailed	19/1225 (1.6%)
Frederick 2020	USA	Cohort study	Retrospective	Esophagectomy; Two-stage (70.5%); Three-stage (21.5%); other (8.0%)	149	Mean follow-up of 26.9 months (Range 0–126.4 months)	Early	Any symptomatic, radiographic or endoscopic evidence of delayed gastric emptying by day 5 post-operatively	114/149 (76.5%)	–	–
Fritz 2018	Germany	Cohort study	Prospective	Esophagectomy; Ivor Lewis (100%)	170	–	Early	Subjective clinical evaluation of DGCE as inpatient	28/170 (16.5%)	–	–
Fuchs 2016	USA	Cohort study	Retrospective	Esophagectomy; Transhiatal (100%)	41	Mean follow-up of 12 months (Range 6–45 months)	Late	–	–	Need for post-operative pyloric intervention	8/41 (8.8%)
Fujimoto 2022	Japan	Cohort study	Retrospective	Esophagectomy; McKeown (100%)	33	100% Follow up to beyond 18 months	Late	–	–	Modified Delphi process consensus	10/33 (30.3%)
Fujita 2012	Japan	RCT	Prospective	Esophagectomy; approach not specified	154	–	Early	Routine upper gastrointestinal contrast swallow study at day 6 post-operatively	13/154 (8.4%)	–	–
Gao 2020	China	Cohort study	Prospective	Esophagectomy; Ivor Lewis (100%)—MIE	34		Early	Selective upper gastrointestinal contrast swallow study during index admission	1/34 (2.9%)	–	–
Giugliano 2017	USA	Cohort study	Prospective	Esophagectomy; Three-stage (77.4%), Ivor Lewis (22.6%)	146	100% Follow up to beyond 6 months	Late	–	–	Need for post-operative pyloric intervention within 6 months	38/146 (26.0%)
Glatz 2017	Germany	Propensity-matched analysis	Retrospective	Esophagectomy; approach not specified	120	–	Late	–	–	Esophagectomy Complications Consensus Group—Timeframe not detailed	20/120 (16.7%)
Godazandeh 2008	Iran	RCT	Prospective	Transhiatal (80.0%), right thoracotomy (20.0%)	30	–	Late	–	–	Radionuclide-labeled gastric scintigraphy at 2 months post-operatively	20/30 (66.7%)
Hadzijusufovic 2019	Germany	Cohort study	Prospective	Esophagectomy; Ivor Lewis (100%)	115	Median follow-up of 16 months (range 4–32 months)	Early and late	Need for post-operative pyloric dilatation before day 14 post-operatively	11/115 (9.6%)	Subjective clinical evaluation of DGCE—Criteria not detailed	21/115 (18.3%)
Hagens 2023	Nether-lands	Cohort study	Prospective	Esophagectomy; approach not specified	159	-	Late	–	–	Esophagectomy Complications Consensus Group—Timeframe within 30 days post-operatively	4/159 (2.5%)
Holscher 2007	Germany	Cohort study	–	Esophagectomy; approach not specified	83	Median follow-up of 7.2 months	Early	Need for post-operative pyloric dilatation during index admission	3/83 (3.6%)	–	–
Huang 2019	China	Cohort study	–	Esophagectomy; Ivor Lewis (100%)—MIE	156	–	Early	Subjective clinical evaluation of DGCE within day 7 post-operatively	49/156 (31.4%)	–	–
Johnson 2019	Australia	Cohort study	Prospective	Esophagectomy; Two-stage (100%)	62	–	Late	–	–	Criteria not detailed	4/62 (6.5%)
Kandagatla 2022	USA	Cohort study	Retrospective	Esophagectomy; Ivor Lewis (100%)	112	–	Late	–	–	Subjective clinical evaluation of DGCE at 12 months post-operatively	3/112 (2.7%)
Kao 1994	Taiwan	RCT	Prospective	Esophagectomy; approach not specified	38	–	Late	–	–	Radionuclide-labeled gastric scintigraphy—Timeframe not detailed	18/38 (47.4%)
Kent 2007	USA	Cohort study	Retrospective	Esophagectomy; approach not specified	12	Median follow-up of 5.3 months	Early and late	Routine upper gastrointestinal contrast swallow study within first week post-operatively	0/12 (0%)	Need for post-operative pyloric intervention	4/12 (8.3%)
Kim 2008	South Korea	Cohort study	Prospective	Esophagectomy; Transthoracic (67.0%); transhiatal (10.0%), transcervical three-field (24.0%)	257	Median follow-up of 26 months	Late	–	–	Radionuclide-labeled gastric scintigraphy—Timeframe not detailed	21/257 (8.2%)
Klevebro 2023	Sweden	Cohort study	Prospective	Esophagectomy; Ivor Lewis (57.1%), McKeown (31.3%), transhiatal (9.8%), left thoracoabdominal (1.8%)—open = 27, MIS = 85	112	–	Early	Routine upper gastrointestinal contrast swallow study at day 2–3 post-operatively	8/112 (7.1%)	–	–
Kuvendjiska 2023	Germany	Cohort study	Retrospective	Esophagectomy; Ivor Lewis (100.0%)—open = 154; hybrid MIE = 152	306	Median follow-up of 21 months	Late	–	–	Criteria not detailed	30/306 (9.8%)
Kuvendjiska 2019	Germany	Cohort study	Retrospective	Esophagectomy; approach not specified	157	–	Early	Routine upper gastrointestinal contrast swallow study at day 5 post-operatively	27/157 (17.2%)	–	–
Lanuti 2011	USA	Cohort study	Retrospective	Esophagectomy; Ivor Lewis (46.0%), Left thoracoabdominal (34.0%), Transhiatal 11.0%), Modified McKeown (3.0%), Minimally invasive (6.0%)	436	–	Early	Routine upper gastrointestinal contrast swallow study at day 4–7 post-operatively	76/436 (17.4%)	–	–
Lee 2005	South Korea	Cohort study	Retrospective	Esophagectomy; approach not specified	56	Mean follow-up of 4.9 months (Range 1–30 months)	Late	–	–	Radionuclide-labeled gastric scintigraphy—timeframe not detailed	21/56 (37.5%)
Lee 2000	Hong Kong	Cohort study	Retrospective	Esophagectomy; Lewis Tanner (55.4%), three-stage (21.5%), esophago-gastrectomy (16.9%), transhiatal (6.2%)	65	Mean follow-up of 84.8 months (Range 10–178 months)	Early	Subjective clinical evaluation of gastroparesis during index admission	3/65 (4.6%)	–	–
Li 2020	China	RCT	Prospective	Esophagectomy; Three-field (50.0%), Two-field (50.0%)	400	100% Follow up to beyond 3 months	Late	–	–	Criteria not detailed	2/400 (0.5%)
Li 2014	China	Cohort study	Prospective	Esophagectomy; left thoracotomy (52.5%), Ivor Lewis (32.0%), McKeown (11.2%), minimally invasive (4.2%)	356	Mean follow-up of 32.3 months (Range 3–66 months)	Early and late	Routine upper gastrointestinal contrast swallow study at day 5–7 post-operatively	26/356 (7.3%)	Clinical and endoscopic evaluation of DGCE 30 days post-esophagectomy—Criteria not detailed	30/356 (8.4%)
Li 2015	China	Propensity-matched analysis	Retrospective	Esophagectomy; cervical-right thoracoabdominal (29.0%), right thoracic–abdominal esophagectomy (16.2%), left thoracic esophagectomy (54.8%)—open = 318, MIE = 89	407	Median follow-up of 27 months (Range 1–99 months)	Late	–	–	Criteria not detailed	7/407 (1.7%)
Lin 2013	China	Cohort study	Prospective	Esophagectomy; approach not specified	150	100% Follow up to beyond 6 months	Late	–	–	Criteria not detailed	4/150 (2.7%)
Liu 2022	China	Cohort study	Retrospective	Esophagectomy; approach not specified	251	-	Late	–	–	Criteria not detailed	2/251 (0.8%)
Liu 2019	China	Cohort study	Retrospective	Esophagectomy; approach not specified	68	100% Follow up to 5 years	Late	–	–	Criteria not detailed	1/68 (1.5%)
Luketich 1998	USA	Cohort study	-	Esophagectomy; Transhiatal (50%), Two-stage (50%)	8	-	Late	–	–	Criteria not detailed	1/8 (12.5%)
Ma 2014	China	Cohort study	Retrospective	Esophagectomy; Sweet (81.7%), Ivor Lewis (18.3%)	915	Median follow-up of 33.6 months (Range 24–72 months)	Early	Selective upper gastrointestinal contrast swallow study or > 400 mL daily nasogastric tube output during index admission	21/915 (23.0%)		
Mannell 1990	South Africa	RCT	Prospective	Esophagectomy; approach not specified	40	Median follow-up of 9 months (Range 2–26 months)	Late	–	–	Subjective clinical evaluation of DGCE/gastric stasis—criteria not detailed	10/40 (25.0%)
Mantoan 2018	Italy	Cohort study	Retrospective	Esophagectomy; Ivor Lewis, McKeown	65	100% Follow up to 3 years	Late	–	–	Need for post-operative pyloric dilatation	9/65 (13.8%)
Marchese 2018	UK	Cohort study	Retrospective	Esophagectomy; Ivor Lewis (100%)	90	–	Early and late	Need for post-operative pyloric dilatation during index admission	4/90 (4.4%)	Need for post-operative pyloric intervention after discharge	6/90 (6.7%)
Margolis 2003	USA	Cohort study	Retrospective	Esophagectomy; approach not specified	80	–	Late	–	–	Criteria not detailed	8/80 (10.0%)
Martin 2009	USA	Cohort study	Prospective	Esophagectomy; approach not specified	45	–	Early and late	Routine upper gastrointestinal contrast swallow study at day 6 post-operatively	4/43 (9.3%)	Subjective clinical evaluation of DGCE at 3 months post-operatively	3/45 (6.7%)
Maus, 2016	Germany	Cohort study	Retrospective	Esophagectomy; approach not specified	403	Study follow-up period of 12 months (Compliance N/S)	Late	–	–	Need for post-operative pyloric dilatation	60/403 (14.9%)
Mehran, 2011	USA	Cohort study	Prospective	Esophagectomy; approach not specified—open = 44, MIE = 44	88	Mean follow-up of 15.2 months (Range 0.8–60.7 months)	Late	–	–	Need for post-operative pyloric dilatation	6/88 (6.8%)
Mertens, 2021	Nether-lands	Cohort study	Prospective	Esophagectomy; approach not specified	239	Median follow-up of 10 months (Range 6–27 months)	Early	Any of the following at 8–10 days post-operatively: (1) daily NGT output > 500 mL; (2) retention of > 300 mL after spigotting NGT for 4 h; (3) Conduit dilatation > 50% compared with index X-ray; (4) Replacement of NGT due to symptoms	15/239 (6.3%)	–	–
Mohajeri, 2016	Iran	RCT	Prospective	Esophagectomy; approach not specified	30	–	Early	Routine upper gastrointestinal contrast swallow study at day 7 post-operatively	18/30 (60%)	–	–
Moons, 2021	Belgium	Propensity-matched analysis	Prospective	Esophagectomy; approach not specified—open = 284; MIE = 168; hybrid = 44	496	–	Late	–	–	Esophagectomy Complications Consensus Group—timeframe not detailed	104/496 (21.0%)
Nafteux, 2011	Belgium	Cohort study	Prospective	Esophagectomy; Approach not specified; MIE = 166	166	86.5% Follow-up to 12 months	Late	–	–	Gastric conduit dilatation on x-ray and need for either prokinetics or pyloric dilatation	19/166 (11.4%)
Nevins, 2020	UK	Cohort study	Prospective	Esophagectomy; Ivor Lewis (75.3%), left thoracoabdominal (20.6%), McKeown (4.1%)	97	–	Early and late	Persistent daily NGT aspirates > 400 mLs from day 3 post-operatively	29/97 (29.9%)	Subjective clinical evaluation of DGCE and gastric conduit dilatation on x-ray at 1 month post-operatively	13/97 (13.4%)
Nevo, 2022	Canada	Cohort study	Prospective	Esophagectomy; Ivor Lewis, McKeown	94	–	Early	Need for NGT re-insertion or inability to remove NGT due to symptoms of DGCE	3/94 (31.9%)	–	–
Nguyen, 2010	USA	Cohort study	Retrospective	Esophagectomy; approach not specified; MIE = 140	140	100% Follow-up to 3 months	Early	Any of the following: (1) > 7 Day requirement for NGT, (2) Gastric conduit dilatation on x-ray, (3) Clinical symptoms of DGCE	7/140 (5.0%)	–	–
Nguyen, 2000	USA	Cohort study	-	Esophagectomy; two-stage (91.7%), transhiatal (8.3%)—MIE	12	Mean follow-up of 12.6 months	Late	–	–	Need for post-operative pyloric intervention	3/12 (25.0%)
Nobel, 2019	USA	Cohort study	Retrospective	Esophagectomy; approach not specified	283	–	Early and late	Persistent daily NGT aspirates > 300 mLs from day 3 post-operatively	201/283 (71.0%)	Need for post-operative pyloric dilatation at 3 months	13/283 (4.6%)
Noshiro, 2007	Japan	Cohort study	Retrospective	Esophagectomy; Approach not specified—MIE	70	Mean follow-up of 32 months	Late	–	–	Criteria not detailed	4/70 (5.7%)
Oezcelik, 2011	USA	Cohort study	Retrospective	Esophagectomy; En bloc transthoracic (66.9%), Transhiatal (30.0%), Minimally invasive (7.1%)	277	Median follow-up of 17 months	Early	Routine upper gastrointestinal contrast swallow study at day 7 post-operatively	33/266 (12.4%)	Subjective clinical evaluation of DGCE—Criteria not detailed	22/277 (7.9%)
Palmes, 2007	Germany	Cohort study	Retrospective	Esophagectomy; abdomino-thoracic (91.4%), Transhiatal (6.6%), cervico-abdomino-thoracic (2.0%)	175	Study follow-up period of 12 months (Compliance N/S)	Early	Routine upper gastrointestinal contrast swallow study at day 4 post-operatively	57/175 (32.6%)	–	–
Park, 2019	South Korea	Cohort study	Retrospective	Esophagectomy; approach not specified	291	–	Late	–	–	DGCE resulting in readmission to hospital—Criteria not detailed	3/291 (1.0%)
Perry, 2009	USA	Cohort study	Prospective	Esophagectomy; thoracoscopic-laparoscopic (42.9%), Transhiatal (57.1%)	70	–	Late	–	–	Criteria not detailed	4/70 (5.7%)
Pines, 2011	Israel	Cohort study	Prospective	Esophagectomy; transhiatal (100.0%)	100	Median follow-up of 19.5 months	Late	–	–	Criteria not detailed	13/100 (13.0%)
Predescu, 2018	Romania	Cohort study	Retrospective	Esophagectomy; approach not specified	82	93.9% Follow-up to 12 months	Late	–	–	Conduit neuro-motor dysfunction—Criteria not detailed	6/82 (7.3%)
Prokakis, 2021	Greece	Cohort study	Retrospective	Esophagectomy; Approach not specified	49	–	Late	–	–	Criteria not detailed	4/49 (8.2%)
Puccetti, 2022	USA	Cohort study	Prospective	Esophagectomy; Ivor Lewis (48.8%), left thoracoabdominal (46.0%), McKeown (9.3%)	43	–	Early	Routine upper gastrointestinal contrast swallow study at day 2–3 post-operatively	7/43 (16.3%)	–	–
Rasmussen, 2021	Denmark	Cohort study	Retrospective	Esophagectomy; Ivor Lewis (100.0%)	120	Median follow-up of 27 months	Late	–	–	Need for post-operative pyloric dilatation	63/120 (52.5%)
Reinstaller, 2022	Germany	Cohort study	Retrospective	Esophagectomy; approach not specified	137	–	Late	–	–	Criteria not detailed	69/137 (50.4%)
Reyhani, 2020	UK	Cohort study	Prospective	Esophagectomy; left thoracoabdominal (100%)—MIE	74	–	Late	–	–	Need for post-operative pyloric dilatation at 6 months	23/74 (31.1%)
Rong, 2022	China	Propensity-matched analysis	Retrospective	Esophagectomy; approach not specified—open = 138; MIE = 132	270	–	Late	–	–	Post-esophagectomy gastroparesis—Criteria not detailed	3/168 (1.8%)
Saeed, 2024	USA	Propensity-matched analysis	Retrospective	Esophagectomy; Ivor Lewis (100%)—robotic, hybrid	475	–	Late	–	–	Both of the following: (1) Presence of one or more DGE-related symptoms; (2) Delayed contrast passage on a barium swallow study	58/475 (12.2%)
Sarkaria, 2019	USA	Cohort study	Prospective	Esophagectomy; Ivor Lewis, Thoracoabdominal, McKeown—open = 106, robotic = 65	170	–	Late	–	–	Post-esophagectomy gastroparesis—Criteria not detailed	1/170 (0.6%)
Schuchert, 2004	USA	Cohort study	Retrospective	Esophagectomy; approach not specified	222	–	Late	–	–	Criteria not detailed	4/222 (1.8%)
Senkowski, 2006	USA	Cohort study	Prospective	Esophagectomy; approach not specified—MIE	20	–	Early	Routine upper gastrointestinal contrast swallow study at day 4–5 post-operatively	2/20 (10.0%)	–	–
Shen, 2022	China	RCT	Prospective	Esophagectomy; approach not specified—MIE	118	–	Late	–	–	Esophagectomy Complications Consensus Group—Timeframe not detailed	1/118 (0.8%)
Shi, 2021	China	Cohort study	Retrospective	Esophagectomy; Ivor Lewis (50.0%), McKeown (50.0%)	136	Median follow-up of 23.5 months (Range 6–36 months)	Late	–	–	Criteria not detailed	2/136 (1.5%)
Skancke, 2017	USA	Cohort study	Retrospective	Esophagectomy; Ivor Lewis (55.6%), transhiatal (44.4%)—open = 18, MIE = 9	27	Study follow-up period of 12 months (Compliance N/S)	Late	–	–	Criteria not detailed	1/27 (3.7%)
Sokouti, 2015	Iran	RCT	Prospective	Esophagectomy; Transhiatal (100.0%)	51	100% Follow up to 12 months	Late	–	–	Radionuclide-labeled gastric scintigraphy at 2 months	19/51 (37.3%)
Stewart, 2017	USA	Cohort study	Prospective	Esophagectomy; Ivor Lewis (96.2%), transhiatal (1.4%), McKeown (1.4%)—MIE	71	100% Follow up to 3 months	Early	Both of the following: (1) Symptoms of DGCE; (2) Features of DGCE on esophagram or upper endoscopy	5/71 (7.0%)	–	–
Sun, 2014	China	Cohort study	Prospective	Esophagectomy; approach not specified—MIE	68	–	Early	Both of the following: (1) Inability to tolerate soft diet by day 10 post-operative; (2) Prolonged gastric emptying time > 25% compared with preoperative radionuclide-labeled gastric scintigraphy	0/68 (0%)	–	–
Sun, 2019	China	RCT	Prospective	Esophagectomy; McKeown (100.0%)—MIE	86	–	Early	Presence of DGCE during index admission—Criteria not detailed	1/86 (1.2%)	–	–
Sutcliffe, 2008	UK	Cohort study	Retrospective	Esophagectomy; transhiatal (58.8%), mckeown (8.5%), left thoraco-abdominal (22.0%), Ivor Lewis (10.7%)	177	Median follow-up of 37 months (Range 1–77 months)	Late	–	–	Both of the following: (1) Symptoms of DGCE; (2) Endoscopic or radiological evidence of gastric distension and pyloric stenosis	21/177 (11.9%)
Swanson	USA	Cohort study	Retrospective	Esophagectomy; transhiatal (80.0%) thoracoabdominal (12.0%), VATS 3-Stage (8.0%)	25	Median follow-up of 22 months (Range 1–84 months)	Early and late	Routine upper gastrointestinal contrast swallow study at week 1–2 post-operatively	3/25 (12.0%)	Need for post-operative pyloric dilatation	1/20 (5.0%)
Takahashi, 2022	USA	Cohort study	Retrospective	Esophagectomy; Ivor-Lewis (57.1%); McKeown (34.6%); transhiatal (8.3%)—MIE	254	–	Early	Both of the following: (1) Symptoms of DGCE within 2 weeks post-operatively; (2) Persistently high nasogastric output or increased gastric conduit dilatation with air-fluid level on x-ray	61/254 (24.0%)	–	–
Tang, 2022	China	Propensity-matched analysis	Retrospective	Esophagectomy; McKeown (37.0%); sweet (56.8%); Ivor Lewis (6.2%)—open = 311; MIE = 175	486	–	Late	–	–	Subjective clinical evaluation of gastroparesis—Criteria not detailed	12/486 (2.5%)
Tapias, 2013	USA	Cohort study	Retrospective	Esophagectomy; Ivor Lewis (45.6%), minimally invasive (8.0%), thoracoabdominal (32.1%), transhiatal (10.3%), modified McKeown (3.8%)	474	Mean follow-up of 43 months	Late	–	–	Criteria not detailed	35/474 (7.4%)
Tham, 2019	UK	Cohort study	Prospective	Esophagectomy; Ivor Lewis (100.0%)	228	–	Early	Persistent daily NGT aspirates from day 5 post-operatively (Inability to remove NGT)	40/228 (17.5%)	–	–
Tham, 2022	UK	Cohort study	-	Esophagectomy; Ivor Lewis (100.0%)	65	100% Follow-up to 6 weeks	Early	Persistent daily NGT aspirates from day 5 post-operatively (Inability to remove NGT)	24/65 (36.9%)	–	–
Uzun, 2024	Germany	Cohort study	Retrospective	Esophagectomy; Ivor Lewis (100.0%)—MIE	439	–	Late	–	–	Criteria not detailed	52/439 (11.8%)
Van der Sluis, 2022	Germany	Cohort study	Prospective	Esophagectomy; Ivor Lewis (86.5%), McKeown (13.5%)—open = 107, hybrid = 101, MIE = 91, RAMIE = 123	422	–	Late	–	–	Esophagectomy Complications Consensus Group—Timeframe not detailed	43/422 (10.2%)
Velanovich, 2003	USA	Cohort study	-	Esophagectomy; approach not specified	58	Mean follow-up of 15 months (Range 1–60 months)	Late	–	–	Subjective clinical evaluation of DGCE—Criteria not detailed	11/58 (19.0%)
Wang, 2021	China	Cohort study	Retrospective	Esophagectomy; McKeown (100.0%)—MIE	108	–	Late	–	–	Criteria not detailed	1/108 (0.9%)
Wang, 2021	China	Cohort study	Prospective	Esophagectomy; McKeown (100.0%)—MIE	192	100% Follow-up to beyond 3 months	Late	–	–	Criteria not detailed	2/192 (1.0%)
Wu, 2022	China	Cohort study	Retrospective	Esophagectomy; modified McKeown (100.0%)	45	–	Late	–	–	Esophagectomy Complications Consensus Group—Timeframe not detailed	0/45 (0.0%)
Xu, 2023	China	Cohort study	Retrospective	Esophagectomy; McKeown (100.0%)—Robotic	211	–	Late	–	–	Criteria not detailed	6/211 (2.8%)
Yajima, 2009	Japan	Cohort study	Retrospective	Esophagectomy; transthoracic (66.0%), transhiatal (34.0%)	141	Median follow-up of 60 months (Range 18–204 months)	Late	–	–	Endoscopic examination for presence of chyme in gastric conduit—Timeframe not detailed	26/141 (18.4%)
Yetasook, 2013	USA	Cohort study	Retrospective	Esophagectomy; Ivor Lewis (100.0%)—Hybrid	24	Median follow-up of 10 months (Range 5–15 months)	Early	Routine upper gastrointestinal contrast swallow study at day 3 post-operatively	6/24 (25.0%)	–	–
Zhang, 2017	China	Cohort study	Retrospective	Esophagectomy; approach not specified—open = 250, MIE = 35	285	100.0% Follow-up to beyond 3 months	Late	–	–	Both of the following: (1) Symptoms of DGCE; (2) Endoscopic or radiological evidence of gastric distension and pyloric stenosis	52/285 (18.2%)
Zhang, 2019	China	RCT	Prospective	Esophagectomy; approach not specified	104	99.0% follow-up to 5 years	Late	–	–	Criteria not detailed	6/104 (5.8%)
Zhang, 2022	China	Propensity-matched analysis	Retrospective	Esophagectomy; Ivor Lewis, McKeown, thoracic-cervical dual-incision, left thoracic esophagectomy	256	Median follow-up of 47.5 months (Range 3–139 months)	Late	–	–	Subjective clinical evaluation of gastroparesis during index admission	3/256 (1.2%)
Zhang, 2017	China	Cohort study	Retrospective	Esophagectomy; Ivor Lewis (100.0%)	185	Mean follow-up of 38.5 months (Range 6–55 months)	Late	–	–	Criteria not detailed	5/185 (2.7%)
Zhao, 2017	China	Cohort study	Retrospective	Esophagectomy; approach not specified—MIE	273	Median follow-up of 35 months	Late	–	–	Criteria not detailed	7/273 (2.6%)
Zhou, 2009	China	Cohort study	Retrospective	Esophagectomy; modified McKeown (100.0%)—hybrid	30	–	Late	–	–	Criteria not detailed	1/30 (3.3%)

The methodological quality of the included studies varied. RCTs assessed using the RoB 2 tool predominantly demonstrated moderate methodological quality, with consistent limitations including inadequate blinding and incomplete follow-up reporting (Electronic Supplementary Table S2). The ROBINS-I tool identified that 77.9% of cohort and case–control studies were low-risk of bias, with a subset exhibiting heightened risk of bias stemming from confounding factors (Electronic Supplementary Table S3). As illustrated in Electronic Supplementary Fig. 1, no publication bias was detected for early DGCE incidence (Egger’s test, *p* = 0.46), whereas asymmetry was identified for late DGCE incidence (Egger’s test, *p* = 0.0012). Although marked heterogeneity may give rise to such asymmetry, publication bias is a possibility. While substantial heterogeneity may account for this asymmetry, it may also indicate potential publication bias.

### Incidence of DGCE

**Fig. 2:**
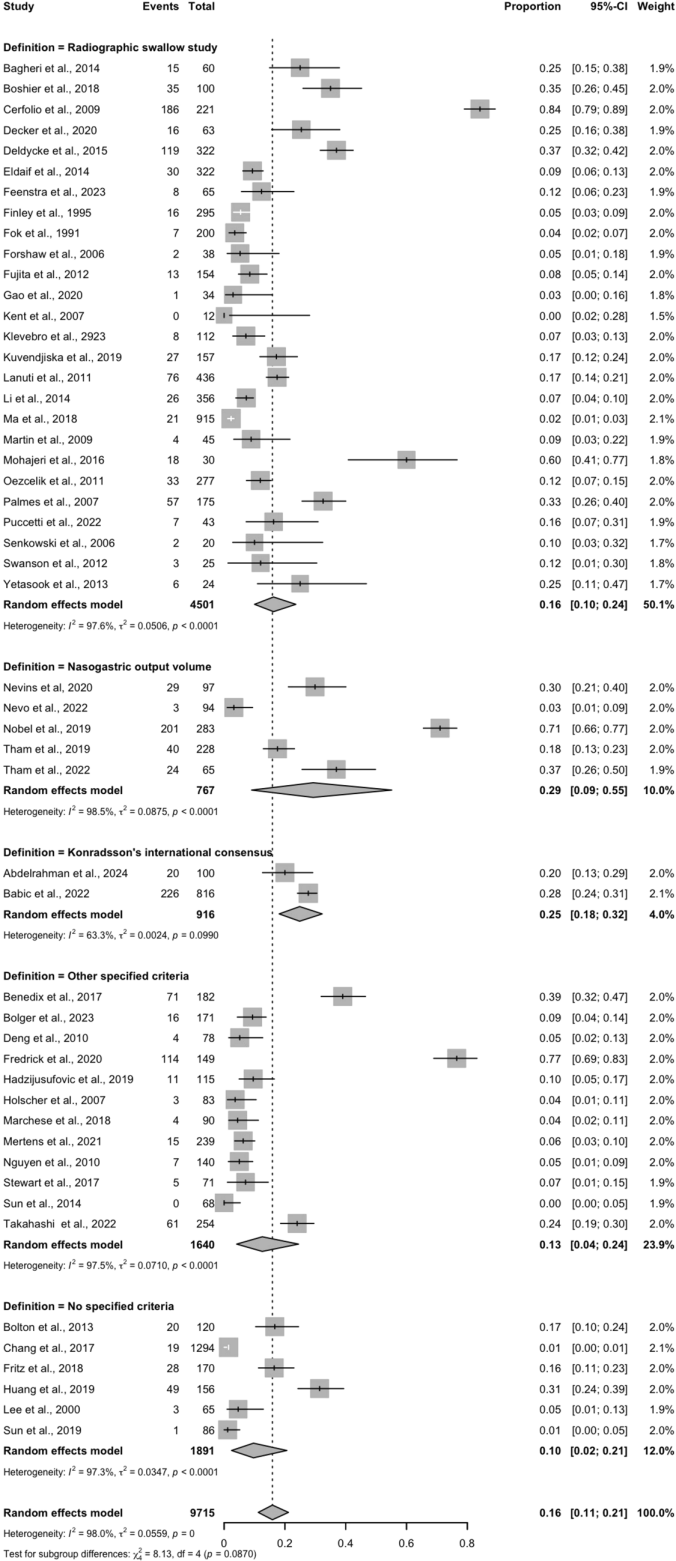
Pooled incidence of early DGCE, categorized by definition

The pooled incidence of early DGCE was 15.9% (95% CI 11.0–21.0%), with significant heterogeneity across studies (*I*^2^ = 97.64%, *p* < 0.001). Definitions of early DGCE varied considerably, with some studies basing their diagnostic criteria solely on radiographic swallow study findings (*n* = 26), nasogastric output volume (*n* = 5), or otherwise adopting Konradsson’s international consensus (*n* = 2). The remaining studies employed either another institution-specific criterion (*n* = 12), as disclosed in Table 1, or did not specify their definition (*n* = 6). Marked heterogeneity persisted within each individual definition group, as evidenced by elevated *τ*^2^ values across all definitional categories. The sole exception was Konradsson’s international consensus (*τ*^2^ = 0.01), though only two studies were included in this group (Fig. 2).

With regards to late DGCE, the pooled incidence was 9.4% (95% CI 7.1–11.9%), also demonstrating high heterogeneity (*I*^2^ = 95.59%, *p* < 0.001). Studies again were wide-ranging in their definition of late DGCE, such as the need for post-operative pyloric intervention to treat delayed emptying symptoms (*n* = 17), assessment via gastric scintigraphy (*n* = 6), the Esophagectomy Complications Consensus Group (ECCG) definition (*n* = 8) [15], or the Konradsson’s international consensus (*n* = 2) [16]. Additional studies employed an independent institution-specific criterion (*n* = 10), while 43 studies did not specify their definition for DGCE. Despite the high overall heterogeneity observed in late DGCE incidence, the analysis within individual definition subgroups demonstrated predominantly low *τ*^2^ values (< 0.1), with the exception of diagnostic criteria based on gastric scintigraphy or post-operative pyloric intervention. This indicates relatively strong agreement in reported incidence rates when consistent diagnostic criteria are applied.

**Fig. 3:**
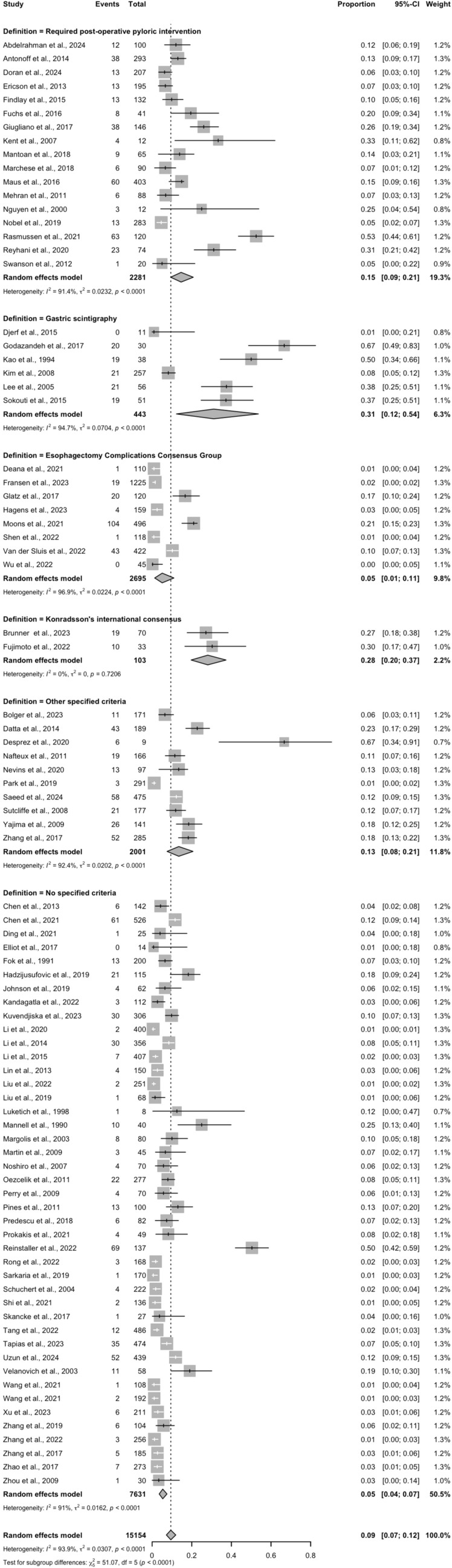
Pooled incidence of late DGCE, categorized by definition

An additional subgroup analysis exploring potential sources of heterogeneity demonstrated that prophylactic pyloric drainage (PPD) was not associated with a statistically significant reduction in early DGCE (OR 0.76; *p* = 0.38) or late DGCE (OR 0.71; *p* = 0.44) (Electronic Supplementary FigS. 3 and 4). This analysis, however, exhibited persistently high *I*^2^ values, indicating considerable residual heterogeneity. Thoracic anastomosis and whole-stomach conduits were associated with marginally higher rates of DGCE, though this association did also not reach statistical significance (Electronic Supplementary Figs. 5–8). Interpretation of these findings was further constrained by the limited number of studies reporting DGCE outcomes stratified by anastomotic height and conduit dimensions (Fig. 3).

## Discussion

This systematic review and meta-analysis provide a comprehensive evaluation of DGCE following esophagectomy, a complication with significant implications for postoperative recovery and quality of life. The pooled incidences of early and late DGCE were 15.9% and 9.4%, respectively, with high heterogeneity observed across studies. These findings highlight the persistent burden of DGCE despite advancements in surgical techniques and perioperative care.

The variability in DGCE definitions across studies underscores a critical challenge in standardizing outcome reporting. Definitions ranged from subjective clinical evaluations to internationally endorsed criteria or standalone diagnostic investigations. Efforts to address this inconsistency were initially spearheaded by the Esophagectomy Complications Consensus Group (ECCG), which brought together 21 high-volume surgeons from 14 countries to develop a set of Delphi consensus definitions for esophagectomy-related complications. DGCE was characterized by the need for intervention or delayed discharge [15]. Konradsson et al. sought to further refine this definition using a modified Delphi process involving a consortium of 33 global experts [16]. This process incorporated both clinical symptoms and radiologic findings to establish diagnostic criteria for early and late DGCE (Table 2), while also introducing a symptom severity grading scale for late DGCE. This framework holds significant promise for improving diagnostic consistency and benchmarking DGCE management. Although only four of the 43 studies published after the publication of Konradsson’s consensus adhered to these criteria [17–20], the limited uptake likely stems from the early stage of adoption of this definition. This gradual dissemination process highlights the inherent challenges of integrating new definitions into established clinical systems and the inconsistent awareness or training among healthcare providers [21]. As survivorship outcomes continue to improve and awareness of this functional issue grows, the adoption of these criteria is anticipated to expand, promoting greater consistency, and standardization in clinical practice.

**Table 2:** International consensus statement on DGCE (adapted from Konradsson et al.)

Diagnostic criteria for early DGCE	One of the following criteria should be fulfilled: > 500 mL Diurnal nasogastric tube output measured on the morning of postoperative day five or later (but within 14 days of surgery); OR > 100% Increased gastric tube width on front chest X-ray projection (in comparison to baseline chest X-ray taken on the day of surgery) together with the presence of an air-fluid level
Diagnostic criteria for late DGCE	Both of the following criteria should be fulfilled: The patient should have “quite a bit” or “very much” of at least two of the following symptoms: Early satiety/fullness Vomiting Nausea Regurgitation Inability to meet caloric need by oral intake Delayed contrast passage on upper gastrointestinal water-soluble contrast radiogram or on timed barium swallow (relying on expert radiologist)

Despite the historical prevalence of contrast swallow studies in the post-operative evaluation after esophagectomy, their notable exclusion from Konradsson’s consensus definition reflects an evidence-based shift in clinical practice [22, 23]. While our review found that over half of the analyzed studies relied on contrast swallow studies as their primary diagnostic tool for early DGCE, mounting evidence suggests this approach has significant limitations. The poor sensitivity and specificity of contrast studies for assessing gastric conduit function and transpyloric emptying, combined with their resource intensity and potential for inconsistent interpretation across institutions, has led many specialized upper gastrointestinal units to move away from their routine use [24–26]. This trend aligns with current ERAS guidelines for esophagectomy [27], which do not mandate contrast swallow studies. The consensus definition’s focus on more objective measures, such as nasogastric tube output and clinical parameters, provides a more robust and practical approach to diagnosing early DGCE [16].

The implementation of standardized diagnostic criteria for DGCE has far-reaching implications for both research and clinical practice. As supported in our analysis, the studies employing explicit diagnostic definitions reported higher incidence rates of DGCE compared to those without defined criteria. This disparity suggests that centers not held accountable to a specific definition are more prone to observer bias, leading to underreporting of DGCE cases. Standardization would not only improve diagnostic accuracy but would also enable meaningful comparisons across institutions and international borders, fostering more robust collaborative research efforts. From a clinical perspective, a uniform criterion also enables early and accurate diagnosis of DGCE, allowing for prompt implementation of tailored interventions to reduce symptom burden and enhance postoperative quality of life.

The subgroup analysis revealed insights into potential modulators of DGCE incidence. Intraoperative pyloric intervention did not confer a statistically significant protective effect against DGCE. Some investigators attribute its suboptimal efficacy to the perioperative localized tissue edema at the pylorus associated with its manipulation [28]. In line with this, the evidence supporting prophylactic pyloric drainage remains equivocal. Several meta-analyses have failed to establish a clear benefit in outcomes from draining the pylorus during esophagectomy [29, 30], with the additional risk of procedural complications introducing substantial uncertainty into the intervention's risk–benefit calculus. Notably, the present subgroup analyses examining prophylactic pyloric drainage continued to demonstrate substantial residual heterogeneity, with persistently high I^2^ values, suggesting that the true sources of heterogeneity could not be fully elucidated despite stratification. The limited efficacy of prophylactic pyloric drainage reflects the nuanced pathophysiology of DGCE, where pyloric dysfunction is significant but not the sole factor determining gastric emptying, particularly given the equally compromised motility of the denervated gastric conduit. Despite the prevailing clinical hypotheses that a more proximal anastomosis and narrow conduit lead to less anatomical redundancy and improved gastric conduit emptying [29, 31–33], neither of these potential sources of heterogeneity demonstrated a statistically significant association. The absence of these findings is in part due to the paucity of studies that compared DGCE incidence data by anastomotic height or conduit diameter. The nuanced interplay between conduit anatomy and DGCE is clearly appreciated by esophagogastric surgeons, however, as more than 80% report constructing a conduit diameter less than 5 cm, and 90% indicate positioning the anastomosis above the azygos vein [34].

A key strength of this study is it allows for more meaningful comparisons across the various definitions of DGCE, spanning multiple institutions and several decades, contributing to a broad and representative understanding of the DGCE burden. Several limitations should also be considered in interpreting this review. The high heterogeneity observed across the included research is a notable drawback, reflecting the variations between studies in both study design and diagnostic criteria for DGCE. Another limitation is that relying on definitions dependent on postoperative pyloric drainage as a surrogate for DGCE may exclude patients who were otherwise managed pharmacologically, potentially underestimating the true incidence of this problem. While the subgroup analysis of anastomosis location may have been constrained by limited study data, potential reporting bias should be considered in its classification. Cervical anastomoses were designated when explicitly documented by authors or when unambiguously described in operative technique details. Given relatively few studies stratified DGCE incidence by anastomotic height, this represents a potential methodological limitation with low statistical power.

## Conclusion

This review demonstrates the significant burden of DGCE following esophagectomy, with considerable variability in reported incidence rates primarily stemming from inconsistent diagnostic definitions. The implementation of standardized criteria is crucial to reduce heterogeneity and mitigate underreporting. Integrating the recently proposed international consensus definition into clinical practice is a step toward enhancing diagnostic accuracy, ultimately improving the identification and management of DGCE. 

## Supplementary Information

Below is the link to the electronic supplementary material.

Supplementary file1 (DOCX 2461 KB)
