# Supplementary material for: Incidence of delayed gastric conduit emptying in patients undergoing esophagectomy: a systematic review and meta‑analysis
Source: Esophagus. 2025 May 28;22(3):289–321. doi: 10.1007/s10388-025-01133-8 (PMC12167245; doi:10.1007/s10388-025-01133-8)
Supplement: Supplementary file 1 — Supplementary file1 (DOCX 2461 KB) [file 10388_2025_1133_MOESM1_ESM.docx]

**Electronic Supplementary Materials - Figure S1. Funnel plot for pooled incidence evaluation of DGCE.**

**
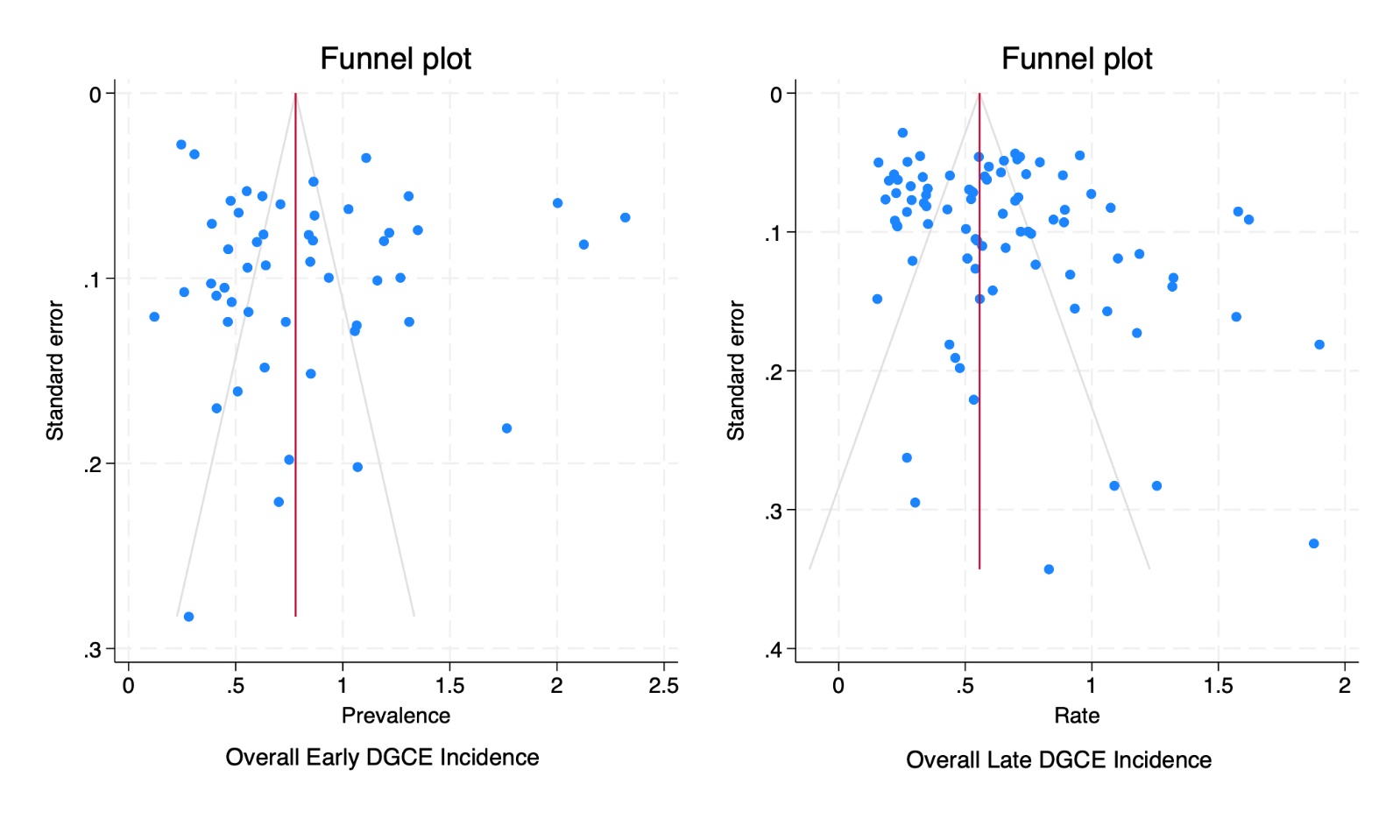
**

**Electronic Supplementary Materials - Figure S2. Effect of prophylactic pyloric drainage on incidence of early DGCE.**


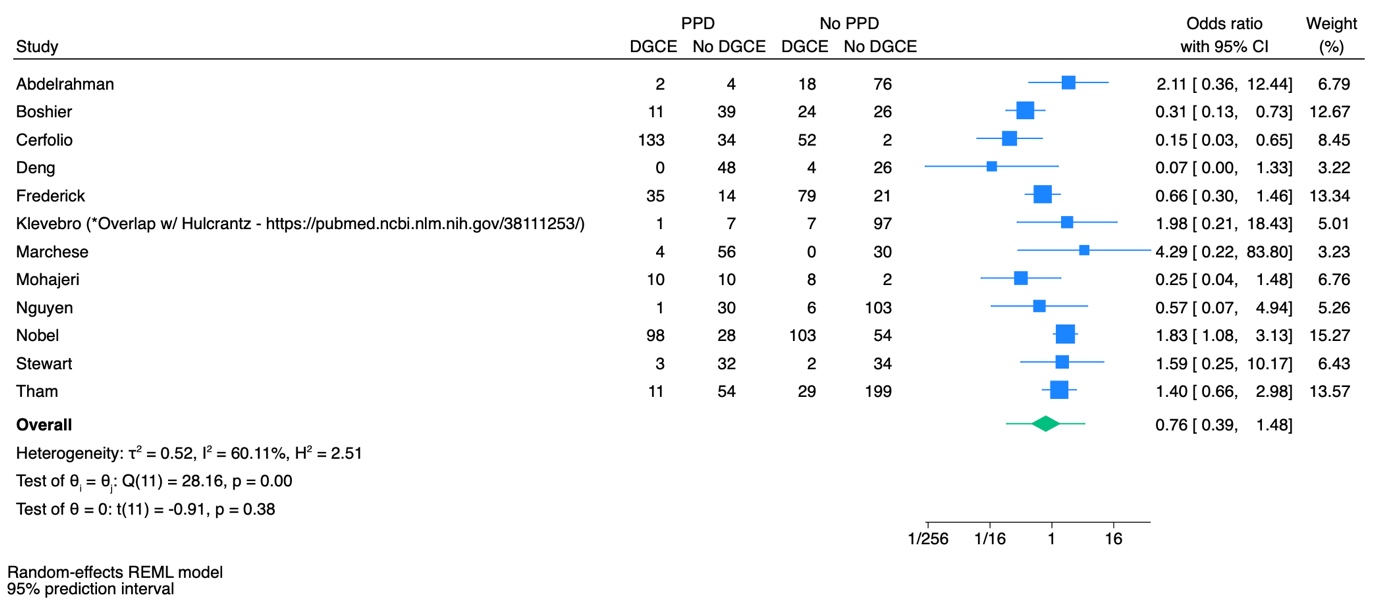


**Electronic Supplementary Materials - Figure S3. Effect of prophylactic pyloric drainage on incidence of late DGCE.**


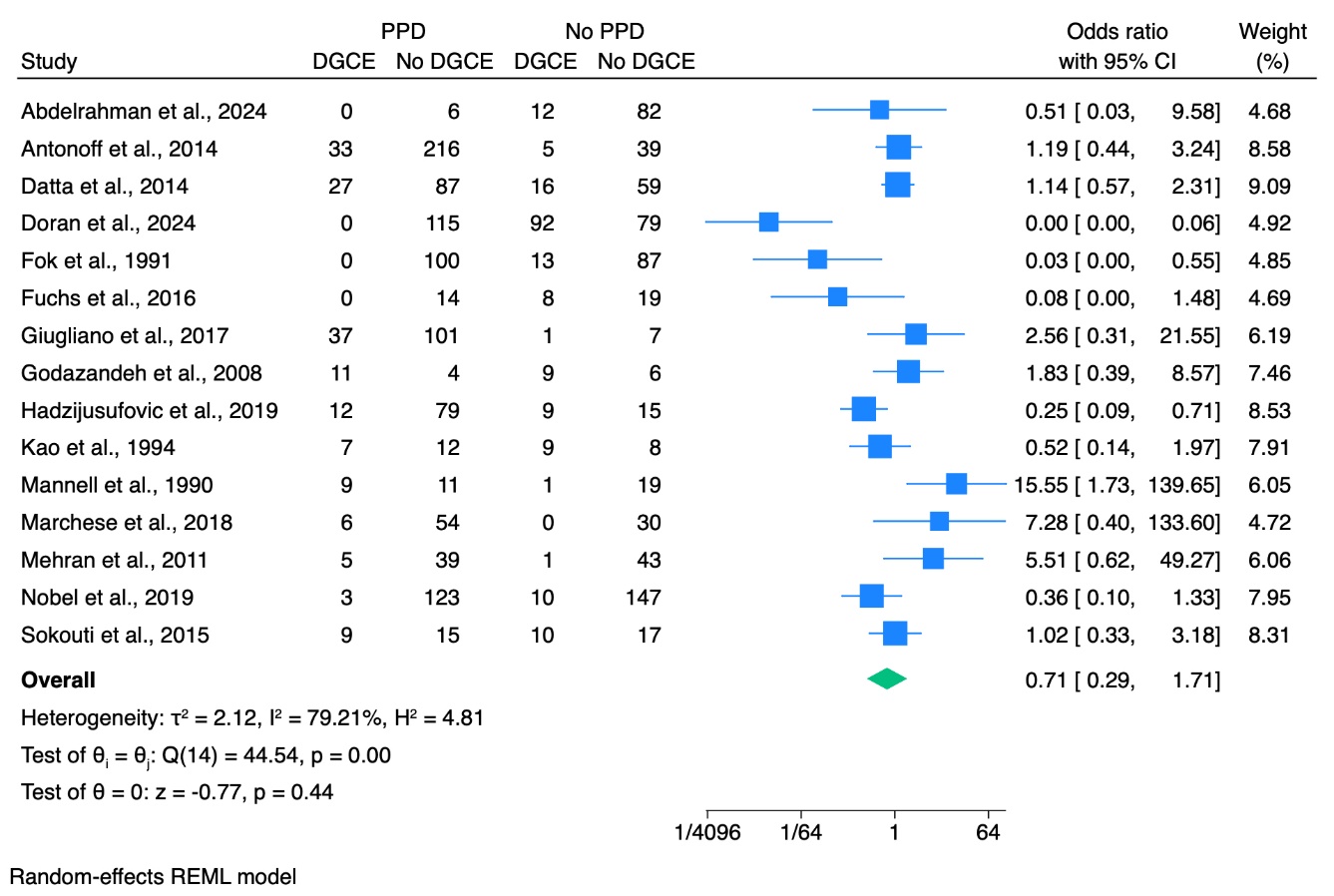


**Electronic Supplementary Materials - Figure S4. Effect of anastomotic height on incidence of early DGCE.**


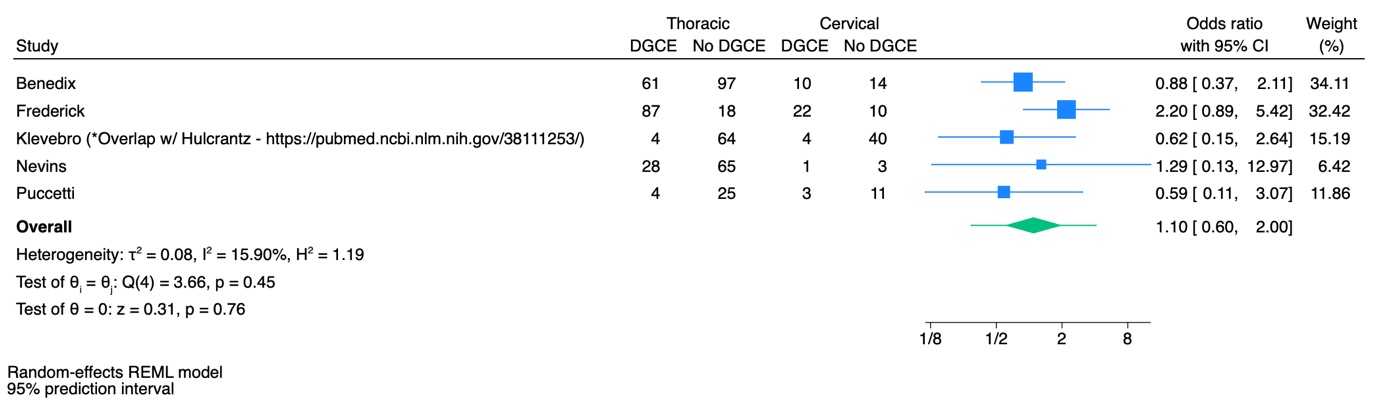


**Electronic Supplementary Materials - Figure S5. Effect of anastomotic height on incidence of late DGCE.**


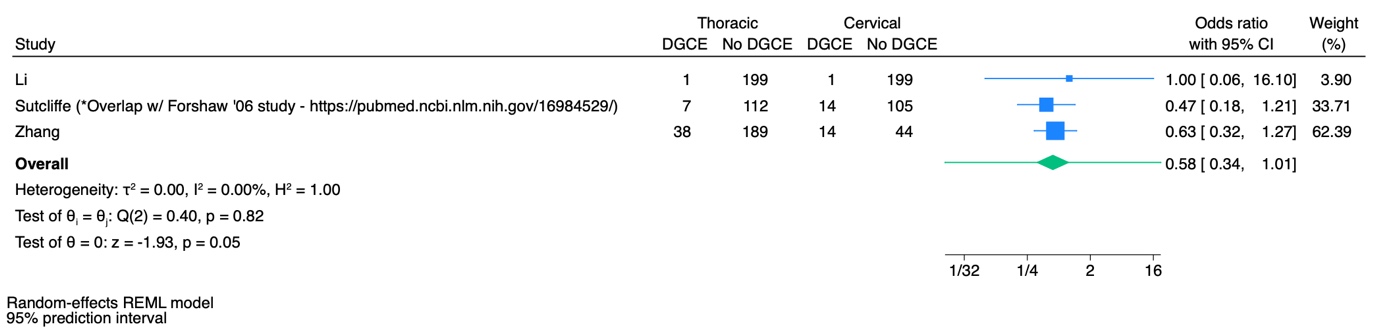


**Electronic Supplementary Materials - Figure S6. Effect of conduit dimension on incidence of early DGCE.**


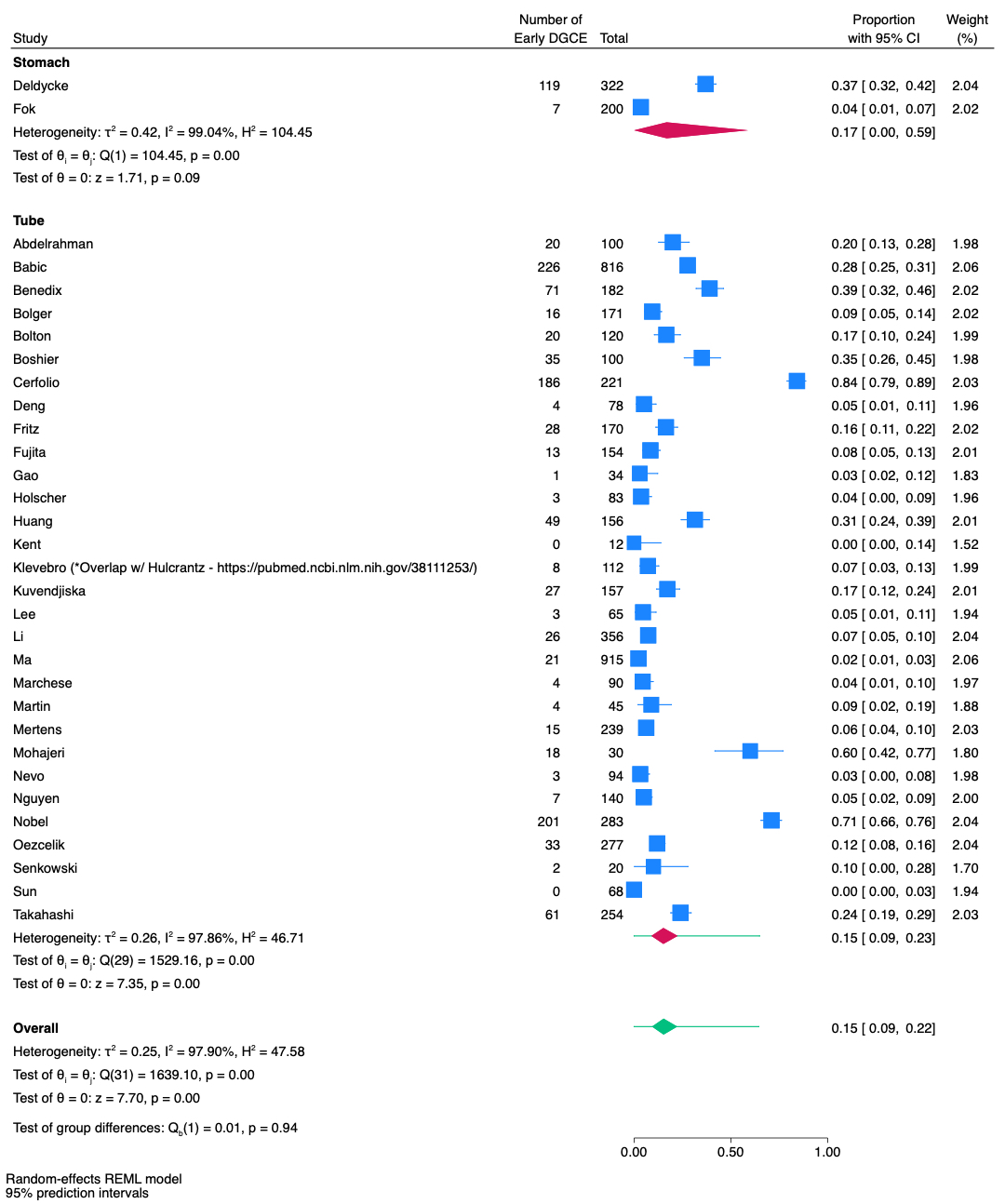


**Electronic Supplementary Materials - Figure S7. Effect of conduit dimension on incidence of late DGCE.**


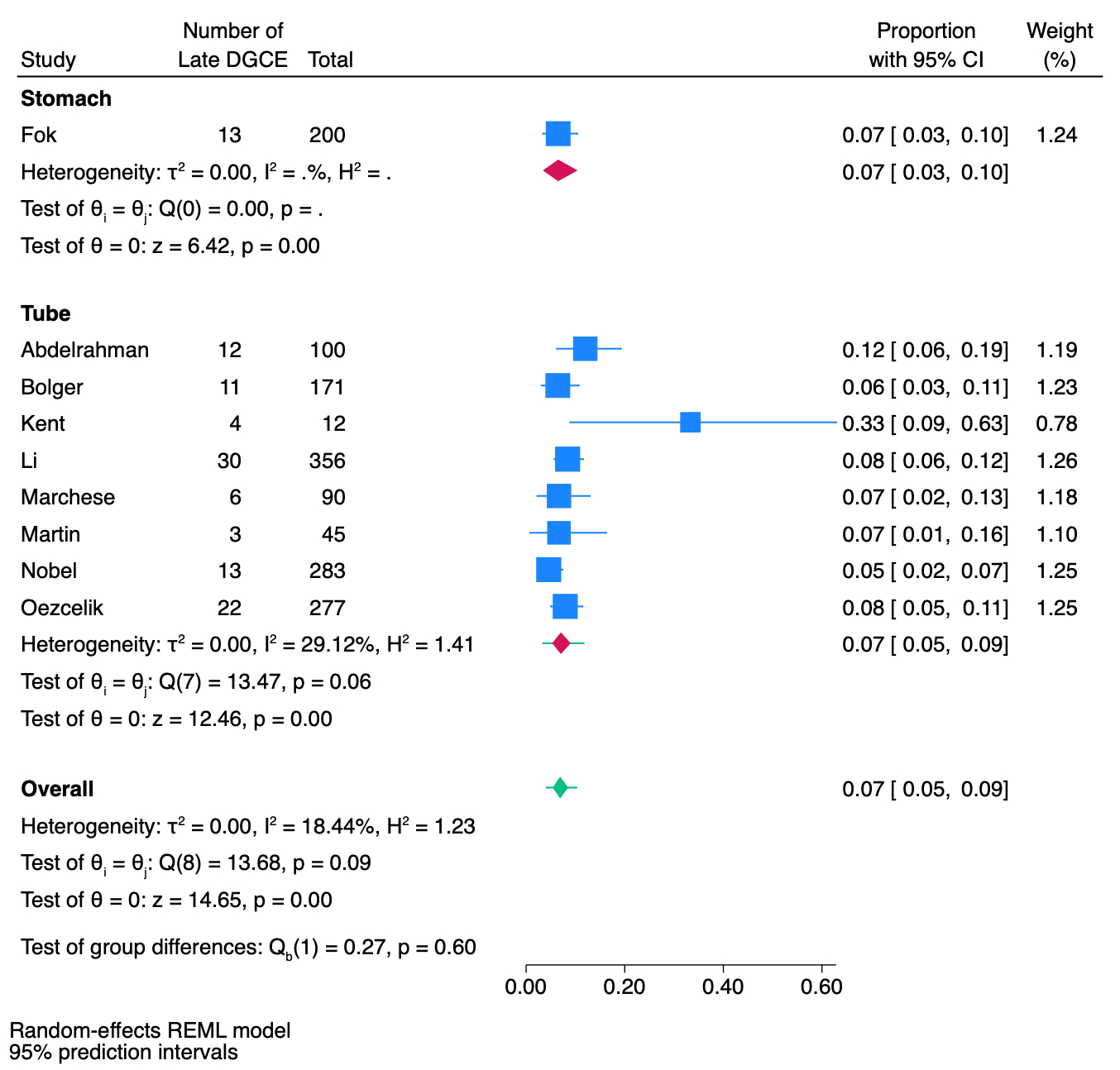


**Electronic Supplementary Materials - Table S1. Search strategy for MEDLINE.**

| **Term groups** | **#** | **Query** | **Hits** |
| --- | --- | --- | --- |
| **Delayed gastric conduit emptying-related terms** | 1 | delayed gastric conduit emptying.mp. | 19 |
|  | 2 | delayed gastric emptying.mp. | 4,105 |
|  | 3 | delayed emptying.mp. | 314 |
|  | 4 | gastric conduit dysfunction.mp. | 3 |
|  | 5 | gastroparesis.mp. | 4,434 |
|  | 6 | functional complications.mp. | 371 |
| **Context-specific terms** | 7 | esophagectomy.mp. | 18,481 |
|  | 8 | oesophagectomy.mp. | 1,913 |
|  | 9 | esophageal cancer surgery.mp. | 665 |
|  | 10 | oesophageal cancer surgery.mp. | 148 |
|  | 11 | esophageal resection.mp. | 1,302 |
|  | 12 | oesophageal resection.mp. | 286 |
|  | 13 | ivor lewis.mp. | 1,084 |
| **Total** | 14 | #2 or #3 or #4 or #5 or #6 | 8,082 |
|  | 15 | #7 or #8 or #9 or #10 or #11 or #12 or #13 | 20,178 |
|  | 16 | #14 and #15 | 162 |
|  | 17 | #1 or #16 | 173 |

**Results from 5^th^ December 2024*

**Electronic Supplementary Materials - Table S2. Quality appraisal using Cochrane Risk of Bias 2.**

| *Study* | *Year* | *Randomisation process* | *Deviations from intended interventions* | *Missing outcome data* | *Measurement of the outcome* | *Selection of the reported result* | *Overall bias* |
| --- | --- | --- | --- | --- | --- | --- | --- |
| *Bagheri* | *2014* | *Low/Some Concerns* | *Low/Some Concerns* | *Low* | *Low* | *Low* | *Low/Some concerns* |
| *Fok* | *1991* | *Low* | *Some Concerns* | *Low/Some concerns* | *Some concerns* | *Low/Some concerns* | *Low/Some concerns* |
| *Fujita* | *2012* | *Low* | *Low* | *Some concerns* | *Low* | *Low* | *Low/Some concerns* |
| *Godazandeh* | *2008* | *High* | *High* | *High* | *High* | *High* | *High* |
| *Kao* | *1994* | *Low* | *Low* | *Some concerns* | *Low* | *Low* | *Some concerns* |
| *Li* | *2020* | *Low* | *Low* | *Low* | *Low* | *Low* | *Low* |
| *Mannell* | *1990* | *Some Concerns* | *High* | *Some concerns* | *High* | *Some concerns* | *High* |
| *Mohajeri* | *2016* | *Low* | *Low* | *Low* | *Low* | *Low* | *Low* |
| *Shen* | *2022* | *Low* | *Low* | *Some concerns* | *Low* | *Low* | *Some concerns* |
| *Sokouti* | *2015* | *Low* | *Low* | *Low* | *Low* | *Low* | *Low* |
| *Sun* | *2019* | *Low* | *Some Concerns* | *Low* | *Low* | *Some concerns* | *Some concerns* |
| *Zhang* | *2019* | *Low* | *Low* | *Some concerns* | *Low* | *Low* | *Low* |

**Electronic Supplementary Materials - Table S3. Quality appraisal using Risk of Bias in Non-Randomized Studies of Interventions.**

| Study | Year | Risk of bias due to confounding | Risk of bias in classification of interventions | Risk of bias in selection of participants into the study (or into the analysis) | Risk of bias due to deviations from intended interventions | Risk of bias due to missing data | Risk of bias arising from measurement of the outcome | Risk of bias in selection of the reported result | Overall |
| --- | --- | --- | --- | --- | --- | --- | --- | --- | --- |
| Abdelrahman | 2024 | Moderate | Low | Low | Low | Low | Low | Low | Low |
| Antonoff | 2014 | Moderate | Low | Low | Low | Moderate | Low | Low | Low |
| Babic | 2022 | Moderate | Low | Low | Low | Moderate | Low | Low | Low |
| Benedix | 2017 | Low | Low | Low | Low | Low | Low | Low | Low |
| Bolger | 2023 | Moderate | Low | Low | Low | Low | Low | Low | Low |
| Bolton | 2013 | Moderate | Low | Low | Low | Low | Low | Low | Low |
| Boshier | 2018 | Moderate | Low | Low | Low | Low | Low | Low | Low |
| Brunner | 2023 | Low | Low | Low | Low | Low | Low | Low | Low |
| Cerfolio | 2009 | Moderate | Low | Low | Low | Low | Low | Low | Low |
| Chang | 2017 | Moderate | Low | Moderate | Low | Low | Moderate | Moderate | Moderate |
| Chen | 2013 | Moderate | Low | Low | Low | Low | Low | Low | Low |
| Chen | 2021 | Moderate | Low | Low | Low | Low | Low | Low | Low |
| Datta | 2014 | Moderate | Low | Low | Low | Low | Low | Low | Low |
| Deana | 2021 | Moderate | Low | Low | Low | Low | Low | Low | Low |
| Decker | 2020 | Moderate | Low | Low | Low | Low | Low | Low | Low |
| Deldycke | 2015 | Moderate | Low | Low | Low | Low | Low | Low | Low |
| Deng | 2010 | Moderate | Low | Low | Low | Low | Low | Low | Low |
| Desprez | 2020 | Low | Low | Low | Low | Low | Low | Low | Low |
| Ding | 2021 | Moderate | Low | Low | Low | Moderate | Low | Low | Low |
| Djerf | 2015 | Low | Low | Low | Low | Low | Low | Low | Low |
| Doran | 2024 | Low | Low | Low | Low | Low | Low | Low | Low |
| Eldaif | 2014 | Moderate | Low | Low | Low | Low | Low | Low | Low |
| Elliot | 2017 | Moderate | Low | Low | Low | Low | Low | Low | Low |
| Ericson | 2013 | Moderate | Low | Low | Low | Low | Low | Low | Low |
| Feenstra | 2023 | Moderate | Low | Low | Low | Low | Low | Low | Low |
| Findlay | 2015 | Moderate | Low | Low | Low | Low | Low | Low | Low |
| Finley | 1995 | Low | Low | Low | Low | Low | Low | Low | Low |
| Forshaw | 2006 | Moderate | Low | Low | Low | Low | Low | Low | Low |
| Fransen | 2023 | Low | Low | Low | Low | Low | Low | Low | Low |
| Frederick | 2020 | Low | Low | Low | Low | Low | Low | Low | Low |
| Fritz | 2018 | Low | Low | Low | Low | Low | Low | Low | Low |
| Fuchs | 2016 | Low | Low | Low | Low | Low | Low | Low | Low |
| Fujimoto | 2022 | Moderate | Low | Low | Low | Low | Low | Low | Low |
| Gao | 2020 | Moderate | Low | Low | Low | Low | Low | Low | Low |
| Giugliano | 2017 | Low | Low | Low | Low | Low | Low | Low | Low |
| Glatz | 2017 | Low | Low | Low | Low | Low | Low | Low | Low |
| Hadzijusufovic | 2019 | Low | Low | Low | Low | Low | Low | Low | Low |
| Hagens | 2023 | Low | Low | Low | Low | Low | Low | Low | Low |
| Holscher | 2007 | Moderate | Low | Low | Low | Low | Low | Low | Low |
| Huang | 2019 | Low | Low | Low | Low | Low | Low | Low | Low |
| Johnson | 2019 | Low | Low | Low | Low | Low | Low | Low | Low |
| Kandagatla | 2022 | Low | Low | Low | Low | Low | Low | Low | Low |
| Kent | 2007 | Moderate | Low | Low | Low | Low | Low | Low | Low |
| Kim | 2008 | Low | Low | Low | Low | Low | Low | Low | Low |
| Klevebro | 2023 | Moderate | Low | Moderate | Low | Moderate | Low | Low | Moderate |
| Kuvendjiska | 2023 | Low | Low | Low | Low | Low | Low | Low | Low |
| Kuvendjiska | 2019 | Moderate | Low | Moderate | Low | Moderate | Moderate | Low | Moderate |
| Lanuti | 2011 | Moderate | Low | Low | Moderate | Low | Low | Moderate | Moderate |
| Lee | 2005 | Moderate | Low | Low | Low | Moderate | Low | Low | Moderate |
| Lee | 2000 | Moderate | Low | Low | Low | Moderate | Low | Low | Moderate |
| Li | 2014 | Moderate | Low | Moderate | Low | Low | Moderate | Low | Moderate |
| Li | 2015 | Low | Low | Low | Moderate | Low | Low | Moderate | Low |
| Lin | 2013 | Moderate | Low | Low | Low | Low | Low | Low | Low |
| Liu | 2022 | Low | Low | Low | Low | Low | Low | Low | Low |
| Liu | 2019 | Moderate | Low | Low | Low | Low | Low | Low | Moderate |
| Luketich | 1998 | Serious | Low | Low | Low | Low | Low | Moderate | Serious |
| Ma | 2014 | Moderate | Low | Low | Low | Low | Low | Low | Low |
| Mantoan | 2018 | Moderate | Low | Low | Low | Moderate | Low | Low | Low |
| Marchese | 2018 | Moderate | Low | Low | Low | Low | Low | Low | Moderate |
| Margolis | 2003 | Moderate | Low | Low | Low | Low | Low | Low | Low |
| Martin | 2009 | Moderate | Low | Low | Low | Low | Low | Low | Moderate |
| Maus | 2016 | Low | Low | Low | Low | Low | Low | Low | Low |
| Mehran | 2011 | Moderate | Low | Low | Low | Low | Low | Low | Moderate |
| Mertens | 2021 | Low | Low | Low | Low | Low | Low | Low | Low |
| Moons | 2021 | Low | Low | Low | Low | Low | Low | Low | Low |
| Nafteux | 2011 | Moderate | Low | Low | Low | Low | Low | Low | Low |
| Nevins | 2020 | Moderate | Low | Low | Low | Low | Low | Low | Moderate |
| Nevo | 2022 | Moderate | Low | Low | Low | Low | Low | Low | Moderate |
| Nguyen | 2010 | Moderate | Low | Low | Low | Low | Low | Low | Low |
| Nguyen | 2000 | Moderate | Low | Low | Low | Low | Moderate | Low | Moderate |
| Nobel | 2019 | Low | Low | Low | Low | Low | Low | Low | Low |
| Noshiro | 2007 | Moderate | Moderate | Low | Low | Moderate | Moderate | Moderate | Moderate |
| Oezcelik | 2011 | Moderate | Low | Low | Low | Low | Low | Low | Low |
| Palmes | 2007 | Low | Low | Low | Low | Low | Low | Low | Low |
| Park | 2019 | Moderate | Low | Low | Low | Low | Moderate | Low | Moderate |
| Perry | 2009 | Moderate | Low | Moderate | Low | Low | Low | Low | Moderate |
| Pines | 2011 | Moderate | Low | Low | Low | Moderate | Low | Low | Moderate |
| Predescu | 2018 | Low | Low | Low | Low | Low | Low | Low | Low |
| Prokakis | 2021 | Moderate | Low | Low | Low | Low | Low | Low | Low |
| Puccetti | 2022 | Moderate | Low | Low | Low | Low | Low | Low | Low |
| Rasmussen | 2021 | Moderate | Low | Moderate | Low | Low | Low | Low | Moderate |
| Reinstaller | 2022 | Low | Low | Low | Low | Low | Low | Low | Low |
| Reyhani | 2020 | Low | Low | Low | Low | Low | Low | Low | Low |
| Rong | 2022 | Moderate | Low | Moderate | Low | Low | Low | Low | Low |
| Saeed | 2024 | Low | Low | Low | Low | Low | Low | Low | Low |
| Sarkaria | 2019 | Moderate | Low | Moderate | Low | Low | Moderate | Low | Moderate |
| Schuchert | 2004 | Moderate | Low | Low | Low | Low | Low | Low | Low |
| Senkowski | 2006 | Moderate | Low | Low | Low | Low | Low | Low | Low |
| Shi | 2021 | Low | Low | Low | Low | Low | Low | Low | Low |
| Skancke | 2017 | Moderate | Low | Low | Low | Moderate | Low | Low | Moderate |
| Stewart | 2017 | Low | Low | Low | Low | Low | Low | Low | Low |
| Sun | 2014 | Low | Low | Low | Low | Low | Low | Low | Low |
| Sutcliffe | 2008 | Moderate | Low | Low | Low | Low | Low | Low | Low |
| Swanson | 2012 | Low | Low | Low | Low | Low | Low | Low | Low |
| Takahashi | 2022 | Low | Low | Low | Low | Low | Low | Low | Low |
| Tang | 2022 | Low | Low | Low | Low | Low | Low | Low | Low |
| Tapias | 2013 | Moderate | Low | Low | Low | Low | Low | Low | Low |
| Tham | 2019 | Low | Low | Low | Low | Low | Low | Low | Low |
| Tham | 2022 | Low | Low | Moderate | Low | Low | Low | Low | Low |
| Uzun | 2024 | Moderate | Low | Moderate | Low | Low | Low | Low | Moderate |
| Van der Sluis | 2022 | Low | Low | Low | Low | Low | Low | Low | Low |
| Velanovich | 2003 | Low | Moderate | Low | Low | Moderate | Low | Low | Low |
| Wang | 2021 | Low | Low | Low | Low | Low | Low | Low | Low |
| Wang | 2021 | Low | Low | Low | Low | Low | Low | Low | Low |
| Wu | 2022 | Moderate | Low | Low | Low | Low | Low | Low | Low |
| Xu | 2023 | Low | Low | Low | Low | Low | Low | Low | Low |
| Yajima | 2009 | Low | Moderate | Low | Low | Low | Moderate | Low | Moderate |
| Yetasook | 2013 | Low | Low | Low | Moderate | Low | Low | Low | Low |
| Zhang | 2017 | Low | Low | Low | Low | Low | Low | Low | Low |
| Zhang | 2022 | Low | Low | Low | Low | Low | Low | Low | Low |
| Zhang | 2017 | Moderate | Low | Low | Low | Low | Low | Low | Moderate |
| Zhao | 2017 | Low | Low | Low | Low | Low | Low | Low | Low |
| Zhou | 2009 | Low | Low | Low | Moderate | Low | Low | Low | Low |
